# Novel Mesoporous and Multilayered Yb/N-Co-Doped CeO_2_ with Enhanced Oxygen Storage Capacity

**DOI:** 10.3390/ma16155478

**Published:** 2023-08-04

**Authors:** Yaohui Xu, Liangjuan Gao, Pingkeng Wu, Zhao Ding

**Affiliations:** 1 Laboratory for Functional Materials, School of New Energy Materials and Chemistry, Leshan Normal University, Leshan 614000, China; xuyaohui1986@163.com; 2 Leshan West Silicon Materials Photovoltaic and New Energy Industry Technology Research Institute, Leshan 614000, China; 3 College of Materials Science and Engineering, Sichuan University, Chengdu 610065, China; lgao87@scu.edu.cn; 4 Department of Chemical Engineering, Illinois Institute of Technology, Chicago, IL 60616, USA; pwu18@hawk.iit.edu; 5 College of Materials Science and Engineering, National Engineering Research Center for Magnesium Alloys, Chongqing University, Chongqing 400044, China

**Keywords:** CeO_2_, doping, solvothermal, oxygen storage capacity, porous

## Abstract

A cubic fluorite-type CeO_2_ with mesoporous multilayered morphology was synthesized by the solvothermal method followed by calcination in air, and its oxygen storage capacity (OSC) was quantified by the amount of O_2_ consumption per gram of CeO_2_ based on hydrogen temperature programmed reduction (H_2_–TPR) measurements. Doping CeO_2_ with ytterbium (Yb) and nitrogen (N) ions proved to be an effective route to improving its OSC in this work. The OSC of undoped CeO_2_ was 0.115 mmol O_2_/g and reached as high as 0.222 mmol O_2_/g upon the addition of 5 mol.% Yb(NO_3_)_3_∙5H_2_O, further enhanced to 0.274 mmol O_2_/g with the introduction of 20 mol.% triethanolamine. Both the introductions of Yb cations and N anions into the CeO_2_ lattice were conducive to the formation of more non-stoichiometric oxygen vacancy (*V*_O_) defects and reducible–reoxidizable Ce^n+^ ions. To determine the structure performance relationships, the partial least squares method was employed to construct two linear functions for the doping level vs. lattice parameter and [*V*_O_] vs. OSC/*S*_BET_.

## 1. Introduction

Cerium oxide (ceria, CeO_2_) and CeO_2_-based binary or multiple composites are typical oxygen storage materials, which have a wide range of applications in VOC abatement, partial alkyne hydrogenation, oxidative dehydrogenation, and water gas shift [1,2,3,4,5,6,7], even in biomedical applications [8]. CeO_2_ is appealing because of its unique structure and intrinsic oxygen vacancy (*V*_O_) defect, which can be rapidly formed and eliminated on the surface of CeO_2_, giving the redox cycle of Ce^4+^ ⇔ Ce^3+^. This is the source of the oxygen storage capacity (OSC), which could be described by Reaction (1) and could also be written using Kroger and Vink notations as Reaction (2) [9]:

(1) $$\mathrm{Ce}O_{2}\overset{\mathrm{Oxygen}\;\mathrm{release}}{\underset{\mathrm{Oxygen}\;\mathrm{storage}}{⇄}}Ce_{1-x}^{4+}Ce_{x}^{3+}O_{2-x/2}V_{O}{}_{x/2}+x/4O_{2}$$

(2) $${\mathrm{Ce}}_{2}O_{3}\;\overset{{2\mathrm{CeO}}_{2}}{⇄}{\;2\mathrm{Ce}}_{\mathrm{Ce}}^{\prime }+3O_{O}^{\times }+V_{O}^{••}$$

Doping CeO_2_ with other metallic cations proved to be efficient in enhancing its OSC [10,11]. Inspired by La/N [12] and Sm/N [13] co-doped TiO_2_ for enhanced photocatalytic activities, Yb/N doping should be a feasible method for modifying the OSC of CeO_2_ in this work. However, no reports had been found on the OSC of cationic and anionic co-doping of CeO_2_. Based on the similarity–intermiscibility theory and the similar ionic radii of the ytterbium cation (Yb^3+^; 0.98 Å) and the cerium cation (Ce^4+^; 0.97 Å), the doping of Yb cations into the CeO_2_ lattice was feasible, which could be supported by previous reports [14,15]. Nitrogen (N) was a favorite and preferred non-metal dopant due to its relatively small ionization energy and similar ionic radii to oxygen (O). However, besides N-doped TiO_2_ [16,17], there have been very limited reports on doping with N anions into the CeO_2_ lattice. Since the O^2−^ anion (1.38 Å) has a larger size than that of the Ce^4+^ cation (0.97 Å) in the ionic crystal of CeO_2_, it was more difficult to be substituted by larger N^3−^ anions (1.46 Å) [18,19]. In our previous work, we successfully synthesized N-doped CeO_2_ using ammonium persulfate as an inorganic nitrogen source [20].

Hence, the modification of CeO_2_ by co-doping with Yb cation and N anion should be an effective method to enhance its OSC. In our strategy, Yb-/N-doped CeO_2_ was prepared by a solvothermal method followed by calcination in air with Yb(NO_3_)_3_∙5H_2_O as a Yb source and triethanolamine (TEA) as a N source. For Yb/N-co-doped CeO_2_ samples, the nominal content of Yb was 5 mol.%, namely, Yb/(Yb + Ce) (mol.%) = 5, while the molar ratio of N was TEA/Ce(NO_3_)_3_∙6H_2_O = 5, 10, 15, 20, 25, and 30 mol.%, and the as-obtained samples were denoted 5%N/5%Yb-, 10%N/5%Yb-, 15%N/5%Yb-, 20%N/5%Yb-, 25%N/5%Yb-, and 30%N/5%Yb-doped CeO_2_, respectively. Moreover, 1~7 mol.% Yb-doped CeO_2_ was synthesized in the absence of TEA, while pure or undoped CeO_2_ was synthesized in the absence of Yb(NO_3_)_3_∙5H_2_O and TEA.

## 2. Experimental Procedure

### 2.1. Starting Materials

Ce(NO_3_)_3_∙6H_2_O (99.95%), Yb(NO_3_)_3_∙5H_2_O (99.9%), and triethanolamine (TEA, ≥99.0%) were supplied by Aladdin Co. Ltd. (Shanghai, China). Ethylene glycol (AR) and ethanol (≥99.7%) were supplied by Chengdu Kelong Chemical Co. Ltd. (Chengdu, China). Distilled water was used in all experiments, and all chemicals were used as received without further purification.

### 2.2. Synthesis of Yb/N-Co-Doped CeO_2_

Yb/N-co-doped CeO2 was prepared by a solvothermal method followed by calcination in air. Typically, 3.8 mmol Ce(NO3)3∙6H2O, 0.2 mmol Yb(NO3)3∙5H2O, and the desired amounts of TEA were dissolved in a 30 mL mixed solution of ethylene glycol and distilled water (10 vol.% H2O) in a 50 mL Teflon bottle. Then, the bottle was sealed in a stainless-steel autoclave, transferred to an electric oven, and maintained at 200 °C for 24 h. After the autoclave naturally cooled to room temperature, the resulting precipitate was washed with distilled water and ethanol and then dried at 60 °C for 24 h. Finally, the Yb and N co-doping CeO2 powders were obtained by following calcination at 500 °C for 2 h in air. For the Yb/N-co-doping CeO2, Yb/(Yb + Ce) (mol.%) = 5, TEA/Ce(NO3)3∙6H2O (mol.%) = 5, 10, 15, 20, 25, and 30 mol.%, the as-obtained samples designated as 5%N/5%Yb-, 10%N/5%Yb-, 15%N/5%Yb-, 20%N/5%Yb-, 25%N/5%Yb-, and 30%N/5%Yb-doped CeO2, respectively.

### 2.3. Synthesis of Yb-Doped CeO_2_

Yb-doped CeO_2_ with different molar concentrations of Yb cations was synthesized using the same procedure as controls, but in the absence of TEA. Typically, appropriate amounts of Ce(NO_3_)_3_∙6H_2_O and Yb(NO_3_)_3_∙5H_2_O with a total of 4.0 mmol were dissolved in a 30 mL mixed solution of ethylene glycol and distilled water (10 vol.% H_2_O) in a 50 mL Teflon bottle. Then, the bottle was sealed in a stainless-steel autoclave, transferred to an electric oven, and maintained at 200 °C for 24 h. After the autoclave naturally cooled to room temperature, the resulting precipitate was washed with distilled water and ethanol and then dried at 60 °C for 24 h. Finally, the Yb-doped CeO_2_ powders were obtained by following calcination at 500 °C for 2 h in air. The as-obtained Yb-doped CeO_2_ powders with different molar concentrations of Yb were labeled as 1%Yb-doped CeO_2_, 2%Yb-doped CeO_2_, 3%Yb-doped CeO_2_, 4%Yb-doped CeO_2_, 5%Yb-doped CeO_2_, 6%Yb-doped CeO_2_, and 7%Yb-doped CeO_2_.

### 2.4. Synthesis of Undoped CeO_2_

Pure or undoped CeO_2_ was synthesized using the same procedure as controls, but in the absence of Yb(NO_3_)_3_∙5H_2_O and TEA. Typically, 4.0 mmol Ce(NO_3_)_3_∙6H_2_O was dissolved in a 30 mL mixed solution of ethylene glycol and distilled water (10 vol.% H_2_O) in a 50 mL Teflon bottle. Then, the bottle was sealed in a stainless-steel autoclave, transferred to an electric oven, and maintained at 200 °C for 24 h. After the autoclave naturally cooled to room temperature, the resulting precipitate was washed with distilled water and ethanol and then dried at 60 °C for 24 h. Finally, the pure CeO_2_ powders were obtained by following calcination at 500 °C for 2 h in air.

### 2.5. Characterization

The crystallographic phases of samples were characterized by X-ray diffraction (XRD, D/MAX 2200 PC, Rigaku, Japan) with 40 kV tube voltages and 40 mA current. The morphologies of samples were evaluated by field-emission scanning electron microscopy (SEM; JEOL–7500F, Tokyo, Japan) with an acceleration voltage of 5 kV. N_2_ adsorption–desorption isotherms were measured using a QuadraSorb SI surface area analyzer (Quantachrome, Boynton Beach, FL, USA), and the BET-specific surface areas were determined using the Brunauer–Emmett–Teller method. The surface composition and binding energy of samples were determined by X-ray photoelectron spectroscopy (XPS, ESCALAB 250Xi, Thermo Scientific, Waltham, MA, USA). The oxygen vacancy defects of samples were characterized using a Raman spectrometer (LabRAM Aramis, Horiba Jobin–Yvon, Paris, France) with a He–Cd laser of 325 nm.

### 2.6. Evaluation of OSC

Hydrogen temperature-programmed reduction (H_2_–TPR) measurements were employed to evaluate the OSC of Yb/N-doped CeO_2_ samples, which were performed using a TP–5080 instrument with a thermal conductivity detector (TCD). Briefly, 0.05 g of sample was pre-treated in a 5 vol.%–O_2_/N_2_ flow (30 mL/min) at 500 °C for 1 h and was cooled down under this flowing O_2_/N_2_. Then, the sample was purged with high-purity N_2_ to remove the excess O_2_ on the surface. Finally, a 5 vol.%–H_2_/N_2_ flow (30 mL/min) was introduced into the reactor, which was heating to ~960 °C (10 °C/min), and the change in H_2_ concentration of the outlet gases was monitored online by TCD.

## 3. Results and Discussion

**Figure 1:**
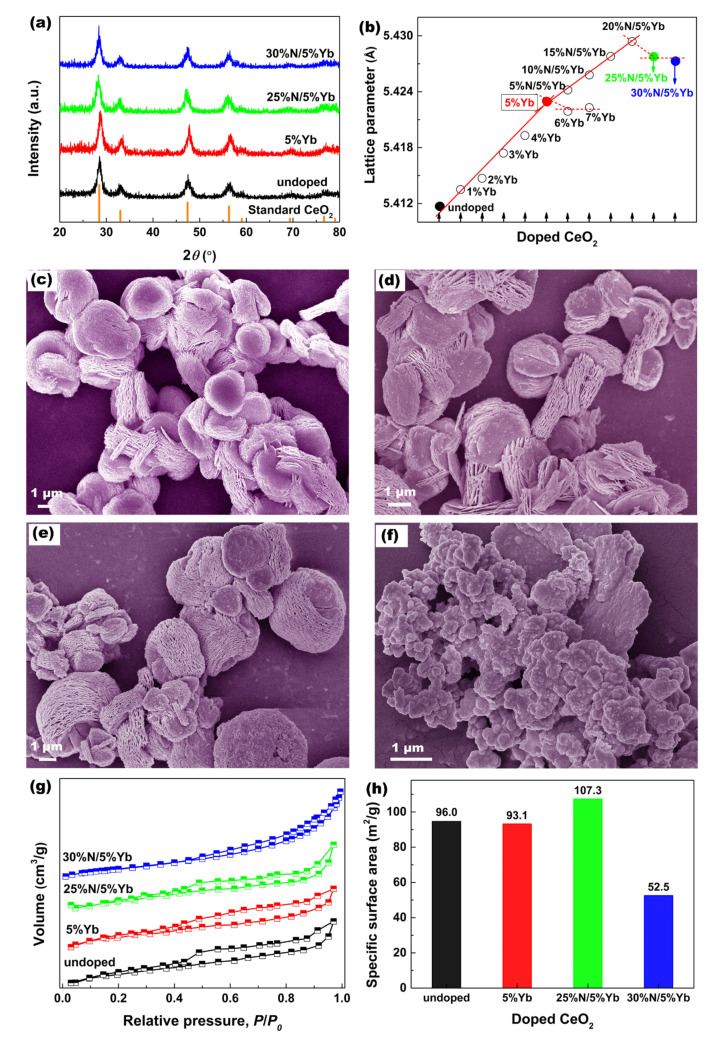
(**a**) XRD patterns of undoped, 5%Yb-doped, 25%N/5%Yb-doped, and 30%N/5%Yb-doped CeO_2_ with standard CeO_2_ (JCPDS No. 34–0349). (**b**) Lattice parameters and fitting curves of Yb/N-co-doped CeO_2_. SEM images of (**c**) undoped, (**d**) 5%Yb-doped, (**e**) 25%N/5%Yb-doped, and (**f**) 30%N/5%Yb-doped CeO_2_. Corresponding (**g**) N_2_ adsorption–desorption isotherms and (**h**) specific surface areas.

Figure 1a shows the XRD patterns of undoped, 5%Yb-doped, 25%N/5%Yb-doped, and 30%N/5%Yb-doped CeO_2_. All identified peaks matched well with the standard CeO_2_ (JCPDS no. 34–0349) pattern, indicating that the CeO_2_ phase with a fluorite structure was obtained, and no other phases (such as CeN, YbN, and Yb_2_O_3_) were detected. Moreover, the corresponding lattice parameters were estimated using Bragg’s equation; the results and the appropriate linear fitting are shown in Figure 1b. The lattice parameters of Yb-doped CeO_2_ and Yb/N-co-doped CeO_2_ were greater than those of undoped CeO_2_, which revealed that the possibly partial substitutions of Ce^4+^ (0.97 Å) and O^2−^ (1.38 Å) ions by the larger Yb^3+^ (0.98 Å) and N^3−^ (1.46 Å) ions happened in the CeO_2_ lattice, and the local lattice distortion (such as lattice expansion) occurred as a result. For Yb-doped CeO_2_, the lattice parameters increased linearly with increasing Yb content until 5%, suggesting that the concentration of Yb in CeO_2_ reached the solid solubility limit with the addition of 5% Yb. Upon the introduction of TEA, the lattice parameters of 5%Yb-doped CeO_2_ continued to increase almost linearly until 20%N, implying that N anions were saturated in the 5%Yb-doped CeO_2_ lattice with the addition of 20%N. In addition, when Yb and N ions reached the solid solubility limit in CeO_2_, and the amount of Yb(NO_3_)_3_∙5H_2_O or TEA continued to increase, their lattice parameters decreased, which indicated that the solid solubility limits of Yb and N in CeO_2_ were supersaturated concentrations. This was attributed to the fact that CeO_2_ itself had a large number of defect structures, leaving CeO_2_ in a metastable state.

SEM was used to study the effect of Yb/N doping on the morphologies of CeO_2_. As seen in Figure 1c, the morphology of undoped CeO_2_ was a multilayered structure consisting of flakes, and these flakes intertwined to form an open porous structure. After the addition of Yb and N ions, the morphologies of 5%Yb-doped CeO_2_ and 25%N/5%Yb-doped CeO_2_ still maintained the multilayered structure, as shown in Figure 1d,e, respectively. Surprisingly, 30%N/5%Yb-doped CeO_2_ in Figure 1f has a completely different morphology from the original multilayered structure. It could be concluded that the addition of TEA had a certain effect on the morphology of CeO_2_. Due to TEA’s alkalescence, excessive TEA addition would destroy the original equilibrium of the self-assembled multilayer morphology in the solvothermal system, which promoted the formation of spheroid particle aggregates.

To further clarify the porous structures of the as-obtained CeO_2_, N_2_ adsorption–desorption experiments were conducted. Figure 1g shows the N_2_ adsorption–desorption isotherms of undoped, 5%Yb-doped, 25%N/5%Yb-doped, and 30%N/5%Yb-doped CeO_2_. All isotherms were consistent with type IV hysteresis loops, confirming their mesoporous structure [21]. The specific surface area (m^2^/g) was an objective, reliable, and physically meaningful size metric for porous materials, and the well-known Brunauer–Emmett–Teller (BET) equation was usually used to determine the specific surface area from the physical adsorption of a gas on a solid surface (labeled as *S*_BET_) [22]. The *S*_BET_ of undoped, 5%Yb-doped, 25%N/5%Yb-doped, and 30%N/5%Yb-doped CeO_2_ powders was estimated and is shown in Figure 1h. The *S*_BET_ of 5%Yb-doped CeO_2_ was almost constant with a value of 93.1 m^2^/g, while the *S*_BET_ value of 25%N/5%Yb co-doped CeO_2_ was 107.3 m^2^/g, slightly higher than that of the undoped sample (96.0 m^2^/g), but the *S*_BET_ value of 30%N/5%Yb-doped CeO_2_ was only 52.5 m^2^/g. It could be concluded that the introduction of TEA had a certain effect on the *S*_BET_, especially when the TEA addition exceeded a certain value. The morphology not only changed significantly, but the *S*_BET_ also decreased sharply.

**Figure 2:**
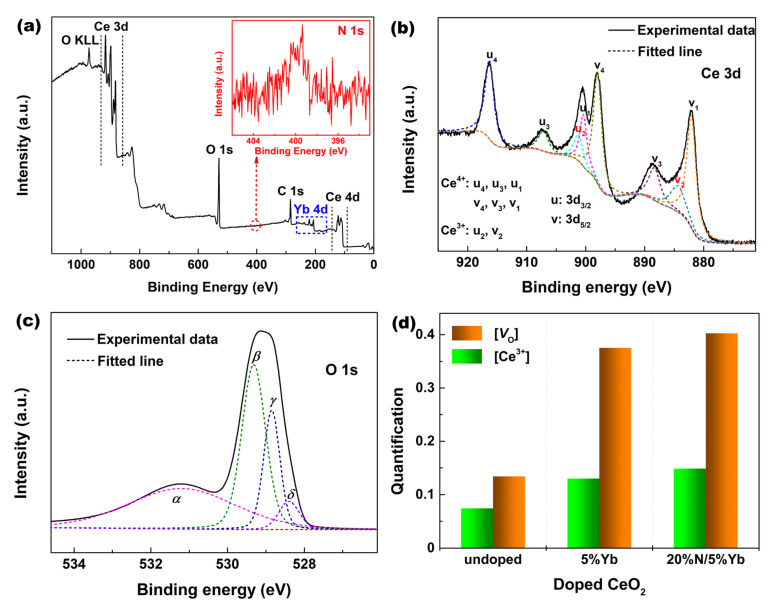
(**a**) Full-range XPS spectrum, (**b**) Ce 3d, and (**c**) O 1s core-level spectra of 20%N/5%Yb-doped CeO_2_ (the inset in Figure 2a is the corresponding N 1s core-level XPS spectrum). (**d**) [Ce^3+^] and [*V*_O_] values of undoped, 5%Yb-doped, and 20%N/5%Yb-doped CeO_2_.

XPS analysis was performed to determine the chemical composition of Yb/N doped CeO_2_ and identify the influence of Yb/N doping on *V*_O_ defects and Ce^n+^ ions in the CeO_2_ crystal. Figure 2a shows the XPS survey spectrum of 20%N/5%Yb-doped CeO_2_. All wide-scan spectra showed clear CeO_2_ features by the signals ascribed to Ce 3d and 4d and O KLL and 1s. Fortunately, the Yb signal could also be detected by the presence of the Yb 4d peak. In addition, the weak peak of the N element could also be clearly observed from the high-resolution XPS spectrum of N 1s in the Figure 2a inset. Figure 2b shows the Ce 3d XPS core-level spectrum of 20%N/5%Yb-doped CeO_2_. The curve of the Ce 3d spectrum was fitted by eight peaks; the bands *u*_4_, *u*_3_, and *u*_1_ (and those for *v*_i_) were attributed to the Ce^4+^ state, while the *u*_2_ and *v*_2_ bands were due to the Ce^3+^ state [23]. Moreover, the O 1s XPS peak of 20%N/5%Yb-doped CeO_2_ is shown in Figure 2c, which was divided into four separate peaks by Gaussian distributions, indicative of the presence of four kinds of oxygen species on CeO_2_. The peaks labeled *β*, *γ*, and *δ* could be assigned to lattice oxygen (*β* for O–Ce^4+^ species, *γ* for O–Ce^3+^ species, and *δ* for O–Yb species), whereas the peak labeled *α* could be assigned to the chemisorption of oxygen or/and weekly bonded oxygen species related to *V*_O_ defects [24].

The relative concentration of Ce^3+^ ions in CeO_2_, labeled as [Ce^3+^], could be calculated by comparing the integrated peak areas of the peak related to Ce^3+^ ions to those of all peaks in Figure 2b. Meanwhile, the relative oxygen vacancy content in CeO_2_ (labeled as [*V*_O_]) could also be estimated from O 1s XPS in Figure 2c by the ratio of the integrated area of the *α* peak to that of all peaks. Figure 2d shows the calculated [Ce^3+^] and [*V*_O_] values of undoped, 5%Yb-doped, and 20%N/5%Yb-doped CeO_2_. Both [Ce^3+^] and [*V*_O_] values of 5%Yb-doped CeO_2_ were higher than those of undoped CeO_2_, which could be explained as follows. Yb^3+^ cations were introduced into the CeO_2_ lattice by the substitution of Ce^4+^ ions, and a substoichiometric CeO_2–*x*_ unit was formed with increasing *V*_O_ defects based on the vacancy compensation mechanism, accompanied by an increase in [Ce^3+^] due to the activation effect of doping. The substitution reaction of Ce^4+^ by Yb^3+^ cations could be written using Kroger and Vink notations as Reaction (3). Moreover, for 20%N/5%Yb-doped CeO_2_, the [Ce^3+^] and [*V*_O_] values were increased further compared with those of 5%Yb-doped CeO_2_, indicating that N anions had been incorporated into the CeO_2_ lattice to form solid solutions, as shown in Reaction (4) using Kroger and Vink notations:

(3) $${\mathrm{Yb}}_{2}O_{3}\;\overset{{2\mathrm{CeO}}_{2}}{⇄}{\;2\mathrm{Yb}}_{\mathrm{Ce}}^{\prime }+3O_{O}^{\times }+V_{O}^{••}$$

(4) $$\mathrm{CeN}\;\overset{{\mathrm{CeO}}_{2}}{⇄}{\;\mathrm{Ce}}_{\mathrm{Ce}}^{\prime }+N_{O}^{\prime }+V_{O}^{••}$$

**Figure 3:**
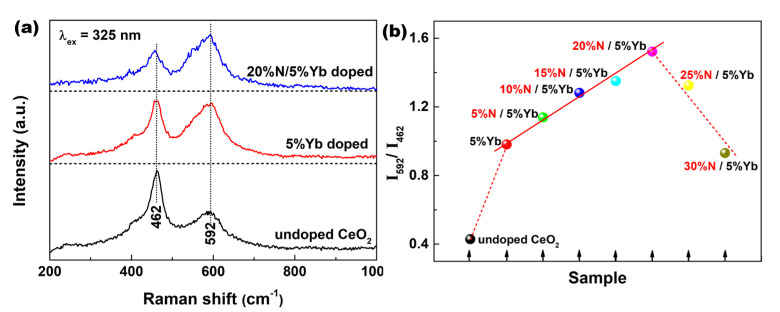
(**a**) Raman spectra of undoped, 5%Yb-doped, and 20%N/5%Yb-doped CeO_2_. (**b**) Relative *V*_O_ concentrations of Yb/N doped CeO_2_ calculated using *I*_592_/*I*_462_ from Raman spectra.

Raman scattering is a very powerful tool for identifying the nature of surface *V*_O_ defects [25]. Figure 3a compares the Raman spectra of undoped, 5%Yb-doped, and 20%N/5%Yb-doped CeO_2_. For undoped CeO_2_, the visible Raman spectrum was dominated by the strong *F*_2g_ mode of the CeO_2_ fluorite phase at ~462 cm^−1^, as well as a weak band at ~592 cm^−1^ assigned to the defect-induced mode, related to *V*_O_ defects. This finding indicated that a certain number of intrinsic *V*_O_ defect sites existed in undoped CeO_2_. Both the band intensities at ~462 and ~596 cm^−1^ had changed with the introduction of Yb and N ions in the CeO_2_ lattice. Typically, compared with the defect-induced band of undoped CeO_2_ at ~592 cm^−1^, that of 5%Yb-doped CeO_2_ had comparable intensity as the *F*_2g_ mode at ~462 cm^−1^. In the case of 20%N/5%Yb-doped CeO_2_, the band intensity of the defect-induced band was even stronger than that of the *F*_2g_ band. Moreover, an alternative approach was provided to estimate the relative concentration of *V*_O_ defects in CeO_2_, namely, the relative number of *V*_O_ defects in CeO_2_ could be determined by the intensity ratio of the bands at ~592 and ~462 cm^−1^, labeled as *I*_592_/*I*_462_. As observed in Figure 3b, with the increasing amount of N in 5%Yb-doped CeO_2_, the relative concentration of *V*_O_ defects, that is, the value of *I*_592_/*I*_462_, increased almost in a straight line and reached a maximum in 20%N/5%Yb-doped CeO_2_. This proved that the additive amount of N anions in fluorite CeO_2_ had an important influence on the formation of *V*_O_ defects in Yb/N-co-doped CeO_2_.

**Figure 4:**
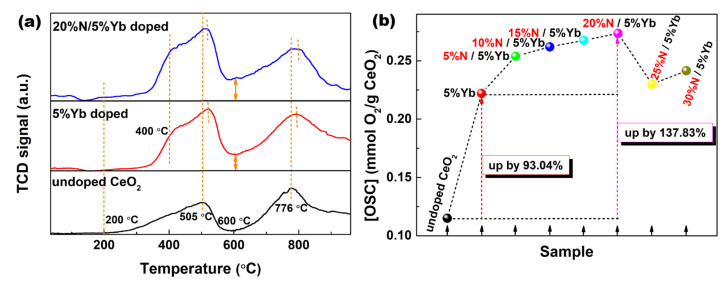
(**a**) H_2_–TPR profiles of undoped, 5%Yb-doped, and 20%N/5%Yb-doped CeO_2_. (**b**) Relative low-temperature OSC of Yb/N-doped CeO_2_.

Figure 4a displays the H_2_–TPR profiles of 5%Yb-doped and 20%N/5%Yb-doped CeO_2_, as well as undoped CeO_2_ for comparison. For the H_2_–TPR spectrum of undoped CeO_2_, the reduction occurred at 200 °C and reached two maximum H_2_ consumptions at ~505 and ~776 °C, implying the existence of at least two kinds of oxygen species at various coordination environments. In other words, the reduction behavior of pure CeO_2_ was divided into two steps: The reduction band at 200~600 °C corresponded to the reduction of surface oxygen species, while the reduction band above 600 °C was assigned to the reduction of bulk oxygen, which could migrate to the CeO_2_ surface through *V*_O_ defects and react with H_2_. Compared to undoped CeO_2_, the 5%Yb-doped and 20%N/5%Yb-doped CeO_2_ could release a certain amount of oxygen below 200 °C, and there appeared to be a visible shoulder from 400 °C in the H_2_–TPR profiles. Moreover, the reduction bands at ~600 °C were far higher than the baseline. These findings indicated that Yb/N doping could effectively improve the OSC of CeO_2_ by increasing the number of *V*_O_ defects. In addition, 5%Yb-doped and 20%N/5%Yb-doped CeO_2_ exhibited a slightly higher redox temperature than that of undoped CeO_2_, which was attributed to the substitutions of Ce^4+^ and O^2−^ with the larger Yb^3+^ and N^3−^ and improved the stability of the CeO_2_ lattice.

OSC is a fundamental characteristic and an important indicator of oxygen storage materials. In CeO_2_-supported catalysts, the porous structure of CeO_2_ is conducive to the loading of other active components, while the excellent OSC favors regulating the oxygen content of the catalytic system. Therefore, the quantification of OSC was the key to comparing their oxygen storage/release properties. For that, the OSC was quantified by the amount of H_2_ consumption per gram of CeO_2_ (mmol H_2_/g CeO_2_) based on H_2_–TPR curves. Finally, the quantified OSC was obtained by calculating the amount of O_2_ released per gram of sample (mmol O_2_/g CeO_2_) according to the H_2_ consumptions, and the quantified OSC at low temperatures is shown in Figure 4b. Compared with the OSC of undoped CeO_2_ (0.115 mmol O_2_/g), that of 5%Yb-doped CeO_2_ reached as high as 0.222 mmol O_2_/g with a 93.04% increase. Upon the introduction of N anions, the OSC of Yb/N-co-doped CeO_2_ continued to increase until 20%N, reached a maximum of 0.274 mmol O_2_/g with a 138.26% increase, decreasing at higher N content. In our previous work, the OSC of CeO_2_ increased by 119.02% and 40.22% upon 5% Hf-doping and 3% Sn-doping, respectively [26]. In our previous study, the OSC of N-doped CeO_2_ microspheres was 0.73 mmol O_2_/g [20]. Among the existing reported OSC data [27,28,29,30,31,32,33], our OSC in this work was above the average level. Even so, the idea of enhancing the OSC of CeO_2_ by cation/anion co-doping has been proven to be feasible in this work.

**Figure 5:**
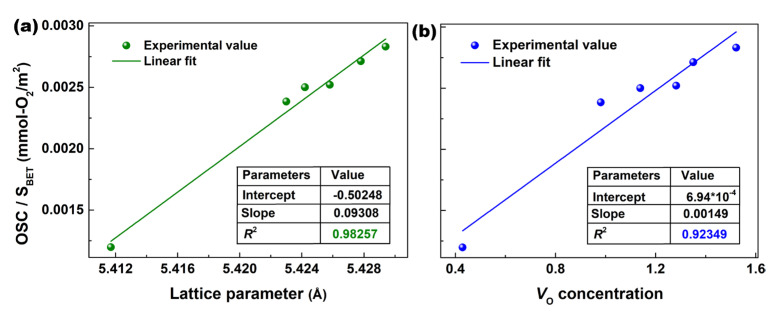
OSC per *S*_BET_ as a function of (**a**) OSC/*S*_BET_ vs. lattice parameter and (**b**) OSC/*S*_BET_ vs. *V*_O_ concentration for Yb/N-co-doped CeO_2_.

*S*_BET_ is an important factor affecting the OSC of CeO_2_, so we developed a function for the OSC/*S*_BET_ vs. lattice parameter as well as the OSC/*S*_BET_ vs. *V*_O_ concentration. These data were linearly fitted based on the partial least squares method, as shown in Figure 5a,b, and the calculated parameters are given as tables in Figure 5a,b. Both Figure 5a,b showed a linear dependence; however, the fitting of the OSC/*S*_BET_ vs. lattice parameter showed a higher correlation coefficient (*R*^2^ = 0.98257) than that of OSC/*S*_BET_ vs. *V*_O_ concentration (*R*^2^ = 0.92349), implying that a larger lattice distortion produced a higher OSC per *S*_BET_ for Yb/N-co-doped CeO_2_. This lattice distortion includes not only the *V*_O_ defects, but also the lattice expansion, as well as the reducible–reoxidizable Ce^n+^ ions, and so on. Thus, the OSC/*S*_BET_ could be predicted as a function of the lattice parameter for Yb/N co-doping CeO_2_.

## 4. Conclusions

In summary, Yb/N-doped mesoporous CeO_2_ with a multilayered structure was synthesized by a solvothermal method using Yb(NO_3_)_3_∙5H_2_O and triethanolamine as cationic and anionic dopants, respectively. Both the original morphologies and fluorite crystal structure of undoped CeO_2_ could be maintained even after the incorporation of 25%N/5%Yb, but the incorporation of Yb/N into CeO_2_ could slightly affect their *S*_BET_. Both the introduction of Yb cations and N anions could cause lattice expansion, accompanied by the formation of more *V*_O_ defects and reducible–reoxidizable Ce^n+^ ions. Compared with the undoped CeO_2_ (0.115 mmol O_2_/g), the OSC of 5%Yb-doped CeO_2_ increased to 0.222 mmol O_2_/g with a 93.04% increase, while that of 20%N/5%Yb-doped CeO_2_ enhanced to 0.274 mmol O_2_/g with a 138.26% increase.

## Data Availability

Not applicable.
